# LCR 5′ hypersensitive site specificity for globin gene activation within the active chromatin hub

**DOI:** 10.1093/nar/gks900

**Published:** 2012-10-05

**Authors:** Kenneth R. Peterson, Halyna Fedosyuk, Susanna Harju-Baker

**Affiliations:** ^1^Department of Biochemistry and Molecular Biology and ^2^Department of Anatomy and Cell Biology, University of Kansas Medical Center, Kansas City, KS 66160, USA

## Abstract

The DNaseI hypersensitive sites (HSs) of the human β-globin locus control region (LCR) may function as part of an LCR holocomplex within a larger active chromatin hub (ACH). Differential activation of the globin genes during development may be controlled in part by preferential interaction of each gene with specific individual HSs during globin gene switching, a change in conformation of the LCR holocomplex, or both. To distinguish between these possibilities, human β-globin locus yeast artificial chromosome (β-YAC) lines were produced in which the ε-globin gene was replaced with a second marked β-globin gene (β^m^), coupled to an intact LCR, a 5′HS3 complete deletion (5′ΔHS3) or a 5′HS3 core deletion (5′ΔHS3c). The 5′ΔHS3c mice expressed β^m^-globin throughout development; γ-globin was co-expressed in the embryonic yolk sac, but not in the fetal liver; and wild-type β-globin was co-expressed in adult mice. Although the 5′HS3 core was not required for β^m^-globin expression, previous work showed that the 5′HS3 core is necessary for ε-globin expression during embryonic erythropoiesis. A similar phenotype was observed in 5′HS complete deletion mice, except β^m^-globin expression was higher during primitive erythropoiesis and γ-globin expression continued into fetal definitive erythropoiesis. These data support a site specificity model of LCR HS-globin gene interaction.

## INTRODUCTION

Globin gene switching continues to be a paradigm for understanding mechanisms of gene regulation during mammalian development (1). In humans, beginning with the embryonic stage of development, continuing through fetal maturation and culminating shortly after birth, the changing needs of the conceptus for oxygen and carbon dioxide exchange require synthesis of different hemoglobin molecules. The protein moieties within the hemoglobin molecule consist of a tetramer containing two β-like globin chains and two α-like globin chains. There are two switches in type of β-like chains utilized and the site of erythropoiesis. The first switch occurs at ∼6 weeks post-conception, when embryonic ε-globin chain expression in the yolk sac changes to fetal γ-globin chain expression in the liver. The second switch begins shortly before birth, where the γ-globin chains are replaced by the penultimate β-globin chain, and to a much lesser extent, the δ-globin chain when the site of erythropoiesis moves to the bone marrow.

The molecular mechanisms controlling expression of the β-like globin gene family members during development have been studied for decades, but much remains to be discovered regarding the underlying regulatory motifs (1–3). The *cis* array of the β-like globin genes is in the order in which they are expressed developmentally, suggesting that gene order affects temporal expression of these genes. In addition to gene-proximal regulatory elements, which are responsible for conferring correct temporal and spatial gene expression upon the cognate genes, the locus control region (LCR) located upstream of the β-like globin gene cluster plays a major role in expression of these genes at the appropriate stage of development (4–7). The LCR activates the β-globin locus (6,7), restricts globin gene expression to the erythroid cell lineage (6,8), enhances globin gene expression (5,6,8) and protects the globin genes from the effects of surrounding negative chromatin (6,9–12).

The LCR is an ∼13-kb sequence located 6–22 kb 5′ to the ε-globin gene. This element comprises five DNaseI-hypersensitive sites (HSs), four are erythroid-specific (5′HS1–4) and one is ubiquitous in many tissues (5′HS5). Each site has a 200- to 300-bp highly conserved core, surrounded by several hundred base pairs to a couple of kilobases of less-conserved sequence similarity (13–16). Each of these cores contains binding sites for erythroid-specific and ubiquitous DNA-binding proteins, generally involved in transcriptional activation or chromatin opening, such as GATA-1, NF-E2 and EKLF (2,3,17–20). Previous data showed that each HS site had a predilection for activation of a specific globin gene or that a combination of several HS sites was necessary for activation of a particular gene, demonstrating HS site specificity played a role in controlling correct developmental regulation of globin gene expression (2,9,10,17,18,21–40). More recent data using chromosome conformation capture (3C) assays (3,41–43) suggest that the LCR folds into a holocomplex, juxtaposing the HS site cores next to each other in three dimensional space to form an active site for delivery of bound co-activators to the developmental stage-specific β-like globin gene promoters. The flanking regions may play a role in determining the folding and maintenance of the DNA–protein structure of the holocomplex (20,23,28). This holocomplex, in turn, is thought to be part of a larger active chromatin hub (ACH; 3,42,44) in which all of the globin genes and the LCR are brought into close proximity in a dynamic active chromatin domain (41). The holocomplex and ACH models, however, do not explain how temporal and site-specific expression is achieved mechanistically.

**Figure 1.**
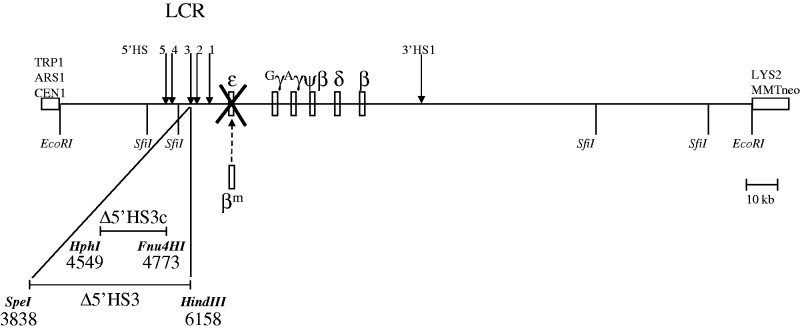
Schematic of Δε::β^m^ β-YAC constructs in transgenic mice. The 2.9-kb ε-globin gene was replaced with a 4.1-kb β^m^-globin gene in wt and LCR 5′HS3-deleted β-YACs as described in the ‘Materials and Methods’ section. wt LCR Δε::β^m^ β-YAC, intact LCR; Δ5′HS3 Δε::β^m^ β-YAC, 2.3 kb LCR 5′HS3 deletion (30); Δ5′HS3c Δε::β^m^ β-YAC, 224 bp LCR 5′HS3 core deletion (28). The β-YAC is indicated as a line with the β-like globin genes shown as boxes with the names of the genes above them. Boxes at the left and right ends are modified pYAC4 vector sequences (48). The location of the β^m^- for ε-globin gene replacement and LCR 5′HS3 deletions are displayed below the line. The LCR 5′HSs, 3′HS1 and YAC/yeast gene components are indicated above the line. TRP1, yeast tryptophan synthesis gene; ARS1, autonomous replicating sequence (yeast origin of replication); CEN1, yeast centromere; LYS2, yeast lysine synthesis gene; MMTneo, mammalian G418-resistance cassette. Restriction enzyme sites are shown below the line and numbered within the human β-globin locus where appropriate (GenBank file U01317).

In this study, we have revisited the function of HS site specificity in globin gene expression and how it might operate in the context of the holocomplex. We designed a set of human β-YAC constructs to distinguish 5′HS3 specificity from LCR conformation as *cis*-acting determinants of β-like globin gene activation (Figure 1). Previously, we showed that LCR 5′HS3 is required for ε-globin gene expression during primitive erythropoiesis and γ-globin gene expression during fetal definitive erythropoiesis in transgenic mice (Supplementary Table S1; 28,30). If LCR conformation, as constrained by the interaction of the HSs within the holocomplex, is the major LCR-related determinant of β-like globin gene expression, then any other developmental stage globin gene replacing the ε-globin gene should not be expressed during primitive erythropoiesis in 5′HS3 mutant β-globin loci, because the LCR would not be in a structural conformation favorable for either ε-globin or alternate β-like globin gene transcription. Conversely, if within the holocomplex 5′HS3 site specificity is the most important *cis*-determinant for directly activating ε-globin gene expression at this developmental stage, then a replacement β-like globin gene should be expressed regardless of the integrity of 5′HS3, because this HS would not be required for expression of other developmental stage β-like globin genes during primitive erythropoiesis. Similar expectations would be predicted for replacement of a γ-globin gene in 5′HS3 mutant loci during fetal definitive erythropoiesis, although the conformation of the LCR or 5′HS3 specificity would be expected to be different from that during primitive erythropoiesis. This specificity change would likely be determined by the difference between the *trans*-acting and epigenetic environments present at these two developmental stages. Our data demonstrate that within the holocomplex, HS site specificity is a determinant of globin gene activation.

## MATERIALS AND METHODS

### β-YAC constructs and transgenic mice

The LCR 5′HS3 complete deletion (5′ΔHS3) and core deletion (5′ΔHS3c) β-YACs were produced as previously described (28,30). A second, marked copy of the β-globin gene (β^m^-globin) was introduced into these β-YACs and the wild-type (wt) β-YAC to replace the ε-globin gene. A 2.9-kb region of the ε-globin gene, containing the silencer, promoter, exons 1, 2 and most of 3 [GenBank coordinates 18 059 (SphI) to 20 958 (BstXI)], was replaced with a 4.1-kb HpaI–XmnI β^m^-globin fragment (GenBank coordinates 61 340 to ∼65 439) derived from pSP73β^m^ (45) by yeast integrating plasmid-mediated homologous recombination in yeast containing the target YACs as previously described (46,47; Figure 1). Murine transgenesis, identification of transgenic mice, copy number determination and structural integrity analysis of the β-YAC molecules in the mice (Supplementary Figures S1–S3) were performed as previously described (46,47).

### Gene expression analysis

RNAse protection assays (RPAs) and semi-quantitative RT–PCR (SQ RT–PCR) reactions were carried out essentially as described (46,47).

## RESULTS

To distinguish 5′HS3 specificity from LCR conformation as *cis*-acting determinants of β-like globin gene expression, we used two separate LCR 5′HS3 deletion mutations linked to an ε- to β-globin gene replacement in human β-globin locus (β-YAC) transgenic mice. We replaced the ε-globin gene with a second, marked copy of the β-globin gene (β^m^-globin) in wt, LCR 5′ΔHS3 and 5′ΔHS3c β-YACs (28,30,48) and used these to produce Δε::β^m^, Δ5′HS3 Δε::β^m^ and Δ5′HS3c Δε::β^m^ transgenic mice, respectively (Figure 1). Three lines containing intact β-globin loci were established for each construct (Supplementary Figures S1–S3). Since the 5′ΔHS3c mutation has the more deleterious effect on globin gene expression between the two 5′HS3 mutations (28,30), we began our analyses with the Δ5′HS3c Δε::β^m^ mice.

**Figure 2.**
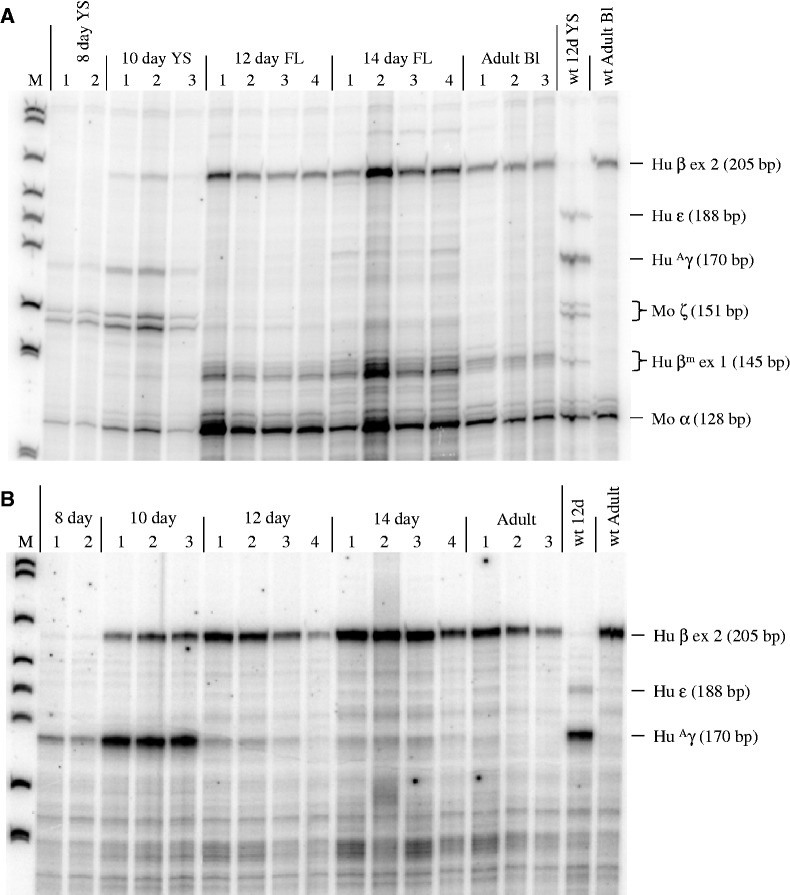
Human β-like globin gene expression during development in Δ5′HS3c Δε::β^m^ β-YAC transgenic mice. (**A**) Hematopoietic tissues. (**B**) Blood. RPA was performed as previously described (46,47). Transgenic line 21 is shown here for illustrative purposes. Individual samples are indicated above the autoradiographs, usually by numbers. Developmental stage (days post-conception), tissue (where appropriate), molecular weight markers (M) and control samples (right-side two lanes of autoradiographs) also are shown. Protected fragments and their sizes are indicated on the right. YS, yolk sac; FL, fetal liver; Bl, blood. Hu β ex 2, human β-globin exon 2; Hu β^m^ ex 1, human β^m^-globin exon 1; Hu ε, human ε-globin; Hu ^A^γ, human ^A^γ-globin; Mo α, mouse α-globin; Mo ζ, mouse ζ-globin.

Human γ- and β-globin and mouse ζ- and α-globin gene expression was measured by RPA in hematopoietic tissues (Figure 2A) and blood (Figure 2B) from developmentally staged Δ5′HS3c Δε::β^m^ conceptuses. β-globin and γ-globin transcription was observed during primitive erythropoiesis in the yolk sac (Figure 2A). During definitive erythropoiesis in the fetal liver, β-globin expression was measurable by RPA, whereas γ-globin gene expression was barely detected. However, γ-globin was clearly observed by a more sensitive SQ RT–PCR assay (data described below and shown in Figures 6 and 7). The transcription pattern of these genes in blood (Figure 2B) reflected the pattern observed in the hematopoietic organs (Figure 2A). These data indicate that 5′HS3 is not required during yolk sac erythropoiesis for either γ- or β-globin synthesis. However, 5′HS3 is necessary for γ-globin expression during fetal liver definitive erythropoiesis, but is not needed for β-globin expression (30).

**Figure 3.**
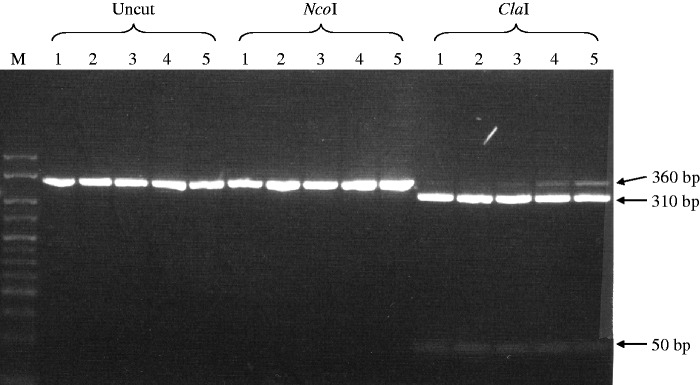
β^m^-globin versus β^wt^-globin gene expression in Δ5′HS3c Δε::β^m^ β-YAC transgenic mice. RT–PCR coupled with restriction enzyme digestion was carried out as previously described (46) to distinguish marked β-globin (β^m^) transcripts from wt β-globin (β^wt^) transcripts. Proof-of-principle data shown are for transgenic line 21. Samples are numbered at the top of the autoradiograph as follows: (1) 8-day yolk sac, (2) 10-day yolk sac, (3) 12-day fetal liver, (4) 14-day fetal liver and (5) adult blood. Uncut, NcoI-digested and ClaI-digested samples also are indicated above the autoradiograph. The uncut PCR product is 360 bp (GenBank HUMHBB 62 138–62 158). NcoI cuts the β^wt^–globin PCR product into 310 and 50 bp fragments. ClaI cuts the β^m^-globin PCR product into 310 and 50 bp fragments. Reciprocal digestion does not occur. Restriction enzyme fragment location and sizes are shown on the right.

The RNAse protection assays were insufficient for distinguishing the β^m^ product from the β^wt^-globin transcripts, so we utilized our previously published RT–PCR protocol coupled with restriction enzyme digests to distinguish the β^wt^-globin product from the β^m^-globin product (46). These assays demonstrated that adult β-globin synthesis during development (Day E8 post-conception through adult) consisted of exclusively or mostly β^m^-globin (Figure 3). NcoI digestion, specific for the wt product, revealed the presence of β^m^-globin only. However, ClaI digestion, selective for the marked product, showed some undigested fragment, indicative of some wt RT product or, alternately, some undigested marked RT product. The NcoI digestion would suggest the later interpretation to be correct, consistent with our previous studies of β-YAC transgenic mice in which the β^m^-globin gene was placed between LCR 5′HS1 and the ε-globin gene (46).

**Figure 4.**
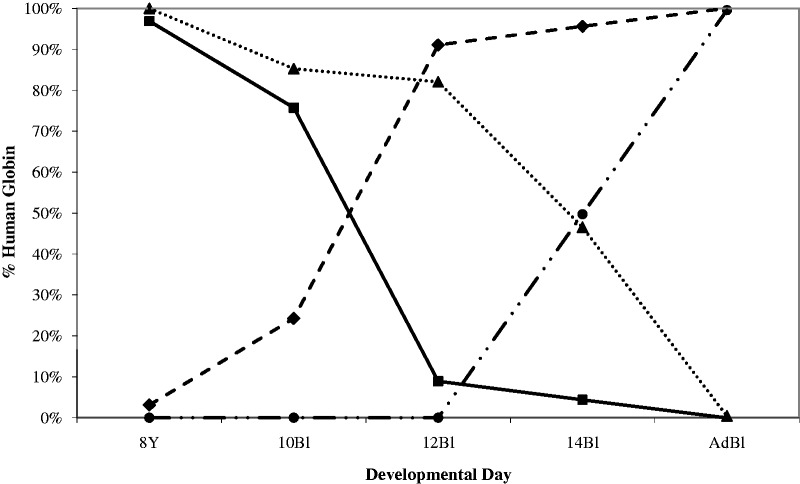
γ- to β-globin gene switching during development in wt and Δ5′HS3c Δε::β^m^ β-YAC transgenic mice. RPA was utilized as described in the ‘Materials and Methods’ section to generate these data. *y*-axis, percent human globin, [γ/(γ+β) × 100]; *x*-axis, developmental day, days post-conception or adult. Square and solid line, γ-globin, Δ5′HS3c Δε::β^m^ β-YAC; diamond and dashed line, β-globin, Δ5′HS3c Δε::β^m^ β-YAC; triangle and dotted line, γ-globin, wt β-YAC; circle and dot-dashed line, β-globin, wt β-YAC.

**Figure 5.**
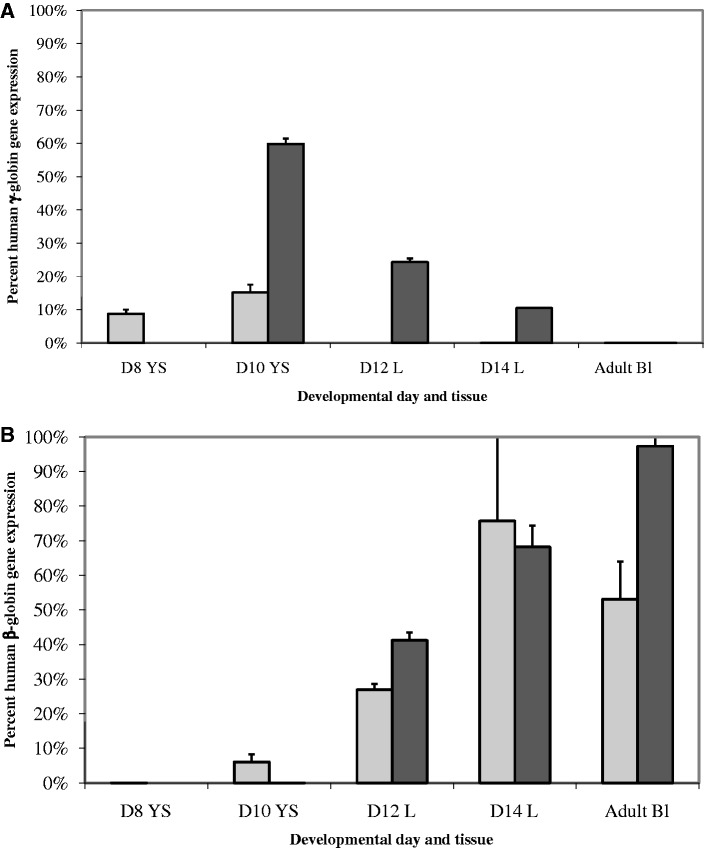
Human β-like globin gene expression during embryonic and definitive erythropoiesis in wt and Δ5′HS3c Δε::β^m^ β-YAC transgenic mice. (**A**) γ-globin.; (**B**) β-globin. Light gray, Δ5′HS3c Δε::β^m^ β-YAC; dark gray, wt β-YAC. RPA was performed as described in the ‘Materials and Methods’ section to produce this data for wt β-YAC line 3547 (48) and Δ5′HS3c Δε::β^m^ β-YAC line 21. *y*-axis, percent Human Globin Gene Expression [(copy number-corrected human γ- or β-globin/copy number-corrected mouse α- + ζ-globin) × 100]; *x*-axis, developmental day and tissue (see Figures 2 and 4 for legend). Data represent the mean and standard error from two to four biological replicates.

The γ-globin to β-globin switch was also completed much earlier during development in Δ5′HS3c Δε::β^m^ mice compared with wt β-YAC mice, essentially when the site of hematopoiesis moved from the yolk sac to the fetal liver between Days E11 and E12 (Figure 4). This observation supports our results above and previous data (30) demonstrating that 5′HS3 is required for γ-globin synthesis during fetal liver definitive erythropoiesis. When the 5′HS3 core is deleted, the switch to β-globin expression is completed rapidly during the transition from embryonic to definitive erythropoiesis. In contrast to published Δ5′HS3c β-YAC lines, where the γ-globin gene was highly expressed in the primitive yolk sac (30), Δ5′HS3c Δε::β^m^ mice express lower levels of γ-globin mRNA (10–15%; Figure 5A), suggesting that the presence of the β^m^-globin gene competitively inhibits γ-globin gene expression. This observation agrees with our previously published experiments, in which we analyzed the effect of gene order on temporal regulation of β-like globin gene expression (46).

To quantify β-like globin gene transcription, we calculated copy-number-corrected γ-globin expression normalized to copy-number-corrected mouse α-globin gene expression confirming the switching profile data; no γ-globin transcription was detected in the fetal liver of Δ5′HS3c Δε::β^m^ mice (Figure 5A). This finding is consistent with the phenotype observed in Δ5′HS3c β-YAC transgenics (30). Copy-number-corrected β-globin transcription normalized to copy-number-corrected mouse α-globin gene expression demonstrated the promiscuous expression of β-globin throughout hematopoiesis, although expression was generally decreased and more variable between samples compared with wt β-YAC samples (Figure 5B), a finding that corroborates data from Δ5′HS3c β-YAC transgenic mice (30).

The next phase of our study sought to address the role of LCR 5′HS3 integrity in supporting ε-globin versus β-globin expression by comparing the various Δε::β^m^ lines carrying Δ5′HS3c, the Δ5′HS3 or the wt LCR compared with LCR variant lines that had a normal ε-globin gene (28,30,48). Our first pass RPA analyses demonstrated that β^m^-globin was expressed in Δ5′HS3c, Δ5′HS3 and wt LCR Δε::β^m^ β-YAC mice beginning on Day 10 yolk sac (Supplementary Figure S4). In addition, γ-globin was co-expressed during embryonic erythropoiesis, but not during fetal definitive erythropoiesis in any of these backgrounds, similar to previous results described earlier [above and (30)].

We utilized SQ RT–PCR to further analyze the β-like globin species that were expressed in various developmentally staged tissues. We expected some variability between SQ RT–PCR and RPA, because of technical differences in the assays. RPA measures the total amount of a queried mRNA species in the sample, because the hybridization reaction is performed in probe excess. Detection is limited by the input amount of total RNA, where the population of any one mRNA may be limiting, below the threshold of detection if the total input is too low. SQ RT–PCR is able to detect mRNA levels below the lowest limits that RPA can measure because of the geometric amplification of the target sequence. Thus, our expectation was that we might see gene expression patterns that differed somewhat between RPA and SQ RT–PCR assays, however, importantly, the data remained consistent between the two data sets.

**Figure 6.**
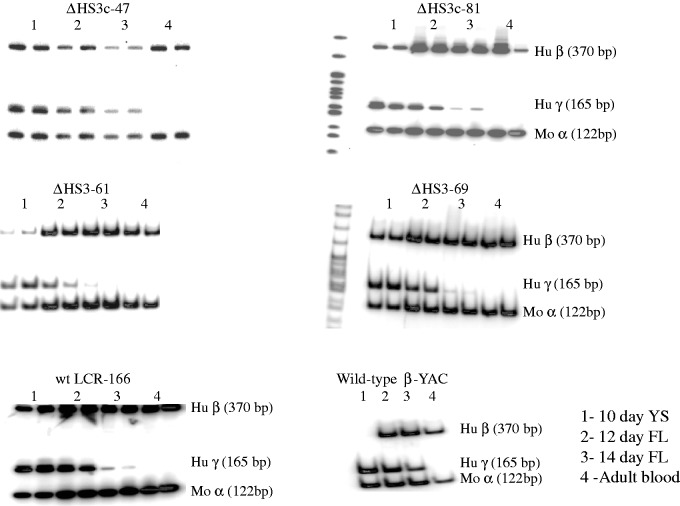
γ- and β-globin gene expression in Δ5′HS3c, Δ5′HS3 and wt LCR Δε::β^m^ β-YAC transgenic mice. Semi-quantitative RT–PCR was utilized to measure transcription as referenced in the ‘Materials and Methods’ section. Top panels, Δ5′HS3c Δε::β^m^ β-YAC transgenic lines; middle panels, Δ5′HS3 Δε::β^m^ β-YAC transgenic lines; bottom panels, wt LCR Δε::β^m^ β-YAC (left) or wt β-YAC (right) transgenic lines. Sample numbering: 1, 10-day yolk sac; 2, 12-day fetal liver; 3, 14-day fetal liver; 4, adult blood. PCR products and sizes are indicated to the right of some panels (Figure 2 for labeling conventions). Data shown are representative experiments used to generate Tables 1–4.

**Figure 7.**
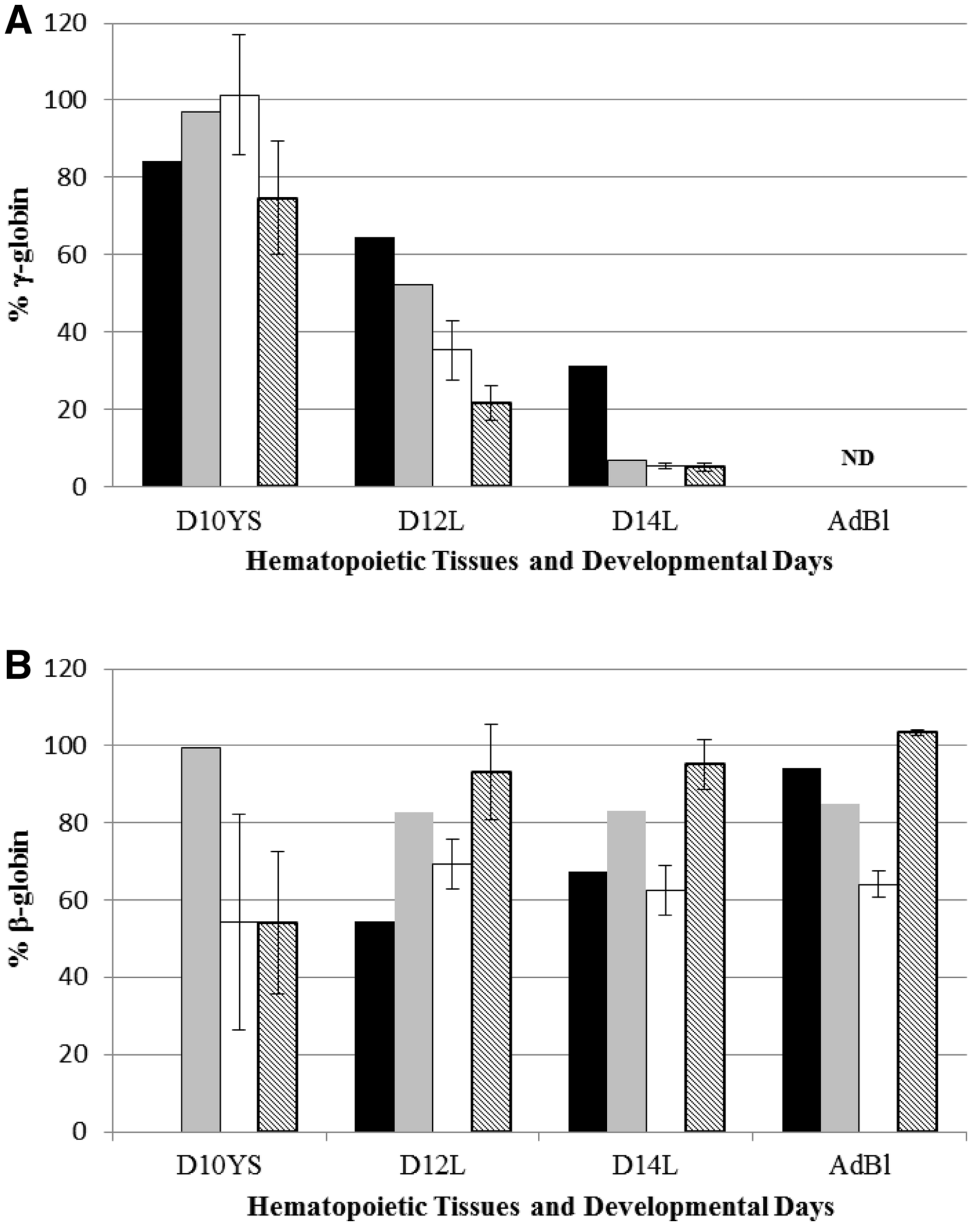
Normalized γ- and β-globin gene expression levels in wt β-YAC, Δ5′HS3c, Δ5′HS3 and wt LCR Δε::β^m^ β-YAC transgenic mice. Averages for each transgenic mouse line from Tables 2 and 4 were averaged and graphed with standard deviations. *x*- and *y*-axes are as described in Figure 5. Black bars, wt β-YAC; gray bars, wt LCR Δε::β^m^ β-YAC; white bars, Δ5′HS3 Δε::β^m^ β-YAC; hatched bars, Δ5′HS3c Δε::β^m^ β-YAC. (**A**) γ-Globin; (**B**) β-globin. γ-Globin gene expression levels between the Δ5′HS3 and Δ5′HS3c lines were analyzed by a two-tailed *t*-test at the different developmental days; no significant differences were found. A two-tailed *t*-test revealed that β-globin gene expression in the Δ5′HS3c lines was significantly higher compared with the Δ5′HS3 lines at Day 14 and adult stage (*P* = 0.011 and 0.0006, respectively).

All Δε::β^m^ lines, regardless of 5′HS3 mutational status, displayed β-globin expression throughout development beginning in the yolk sac at Day 10 (Figure 6), and γ-globin expression in the yolk sac and early in the developing fetal liver (Days 12 and 14), whereas β-globin was not expressed on Day 10 yolk sac from wt β-YAC mice. The qualitative data in Figure 6 show near-normal levels of γ-globin transcripts on Day 10 yolk sac and reduced levels in fetal liver of both the Δ5′HS3c and Δ5′HS3 mice compared with the normal LCR control and wt β-YAC control. Quantitative analysis of the these data [γ/(γ + β^m^ + β^wt^); Table 1] supports the qualitative observations regarding γ-globin gene expression. All of the Δε::β^m^ lines display ∼50% reduction in γ-globin during embryonic erythropoiesis, regardless of their LCR structural status, possibly due to competition between β^m^- and γ-globins for interaction with the LCR, which does not occur in wt β-YAC lines. The effect of the 5′HS status was revealed during early definitive fetal liver erythropoiesis (Day 12), where the Δ5′HS3c mutation had a much more deleterious effect on γ-globin expression than in wt LCR Δε::β^m^, Δ5′HS3 Δε::β^m^ or wt β-YAC lines. At a later stage of fetal liver definitive erythropoiesis (Day 14), the status of 5′HS3 integrity did not influence γ-globin expression. Per copy γ-globin gene expression normalized to mouse α-globin gene expression confirmed this outcome (Table 2 and Figure 7A). This outcome is consistent with our previous studies on Δ5′HS3 and Δ5′HS3c single mutant β-YAC transgenic lines (28,30). Competition between γ- and β-globin genes is leveled at this developmental stage between the Δε::β^m^ lines and the wt β-YAC lines, because γ-globin competes with β^wt^-globin in wt β-YAC lines and with the β^wt^- and β^m^-globins in Δε::β^m^ β-YAC lines. Thus, the expression phenotype is a direct effect of the 5′HS3 genotype.

**Table 1.** γ-globin gene expression as a function of total human globin gene expression during development

Construct	Line no.	YAC copy no.	Embryonic erythropoiesis D10 YS (%)	γ/(γ + β^m ^+ β^wt^)		Adult Bl (%)
				D12 L (%)	Definitive erythropoiesis D14 L (%)	
Δε::β^m^ wt LCR	166	1	54.9 ± 0.6	37.4 ± 3.5	6.3 ± 4.2	0.0
Δε::β^m^ ΔHS3	44	1	57.8 ± 0.9	37.4 ± 7.0	8.7 ± 0.8	0.0
	61	1	71.8 ± 7.7	26.6 ± 4.4	10.5 ± 2.2	0.0
	69	2	53.0 ± 1.8	33.2 ± 5.3	4.1 ± 3.9	0.0
Δε::β^m^ ΔHS3c	47	1	53.0 ± 0.9	19.8 ± 3.5	8.6 ± 0.6	0.0
	81	2	65.5 ± 4.9	18.5 ± 2.5	5.5 ± 2.1	0.0
wt β-YAC average		3	93.2 ± 5.1	48.6 ± 5.5	37.1 ± 6.8	0.0

**Table 2.** Per copy γ-globin gene expression normalized to Mo α-globin gene expression during development

Construct	Line no.	YAC copy no.	Embryonic erythropoiesis D10 YS (%)	γ-globin mRNA		Adult Bl (%)
				D12 L (%)	Definitive erythropoiesis D14 L (%)	
Δε::β^m^ wt LCR	166	1	97.0 ± 2.7	52.1 ± 10.3	6.8 ± 3.1	0.0
Δε::β^m^ ΔHS3	44	1	98.9 ± 3.2	37.3 ± 2.9	5.3 ± 1.1	0.0
	61	1	87.2 ± 8.9	26.7 ± 10.8	6.2 ± 1.7	0.0
	69	2	118.1 ± 13.2	41.8 ± 1.9	4.7 ± 0.9	0.0
Δε::β^m^ ΔHS3c	47	1	64.4 ± 11.5	18.4 ± 1.7	5.6 ± 1.1	0.0
	81	2	85.0 ± 14.5	24.8 ± 7.5	4.2 ± 1.7	0.0
wt β-YAC average		3	84.4 ± 8.1	64.5 ± 3.5	31.2 ± 5.4	0.0

**Figure 8.**
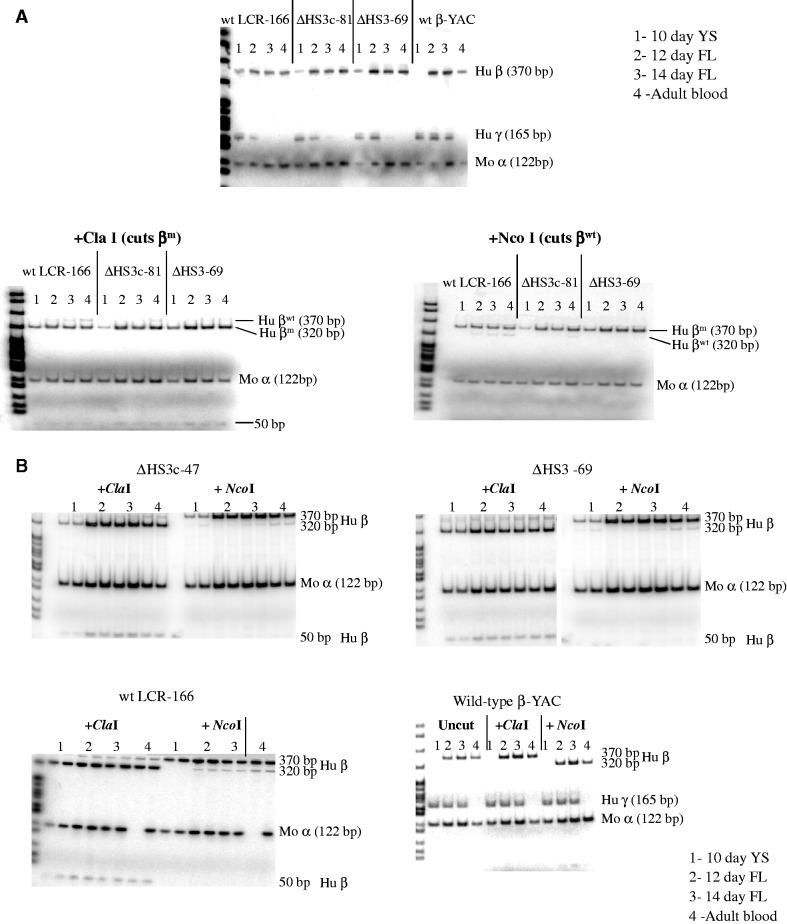
β^m^- and β^wt^-globin gene expression in Δ5′HS3c, Δ5′HS3 and wt LCR Δε::β^m^ β-YAC transgenic mice. Semi-quantitative RT–PCR coupled with restriction enzyme digestion was used as outlined in the legends to Figures 3 and 7. Labeling conventions are also the same as for those figures. Panels **A** and **B** are two representative experiments to show sample data employed to generate Tables 1–4. Sample numbering: 1, 10-day yolk sac; 2, 12-day fetal liver; 3, 14-day fetal liver; 4, adult blood.

Both qualitative and quantitative analyses demonstrated that β-globin expression throughout development consisted of >95% β^m^-globin, and nominal, if any, β^wt^-globin in all of the Δε::β^m^ lines (Figure 8A, B and Table 3). Most importantly, β^m^-globin was robustly expressed regardless of the integrity of 5′HS3 in the Δε::β^m^ lines, as demonstrated by per copy β-globin gene expression normalized to mouse α-globin gene expression (Table 4 and Figure 7B). These data are in stark contrast to ε-globin expression in 5′HS3-compromised β-YAC lines (28,30), supporting our hypothesis that 5′HS3 has a predilection for selectively enhancing ε-globin gene expression during primitive erythropoiesis. Thus, both the RPA and SQ RT–PCR data are consistent with the hypothesis that 5′HS3 shows specificity for ε-globin gene expression in the yolk sac and γ-globin expression in the fetal liver.

**Table 3.** β^wt^-globin gene expression as a function of total human β-globin gene expression during development

Construct	Line no.	YAC copy no.	Embryonic erythropoiesis D10 YS (%)	β^wt^/(β^wt^ + β^m^)		Adult Bl (%)
				D12 L (%)	Definitive Erythropoiesis D14 L (%)	
Δε::β^m^ wt LCR	166	1	4.5 ± 1.4	5.7 ± 0.9	8.4 ± 1.3	12.3 ± 0.6
Δε::β^m^ ΔHS3	61	1	3.8 ± 3.0	3.2 ± 1.1	4.7 ± 1.5	6.9 ± 1.4
	69	2	0.0	8.3 ± 0.1	11.9 ± 1.1	14.5 ± 1.7
Δε::β^m^ ΔHS3c	47	1	0.0	2.9 ± 0.4	3.6 ± 0.3	5.2 ± 0.6
	81	2	0.0	2.6 ± 0.0	3.1 ± 0.7	5.4 ± 0.7

**Table 4.** Per copy β-globin gene expression normalized to Mo α-globin gene expression during development

Construct	Line no.	YAC copy no.	Embryonic erythropoiesis D10 YS (%)	β-globin mRNA		Adult Bl (%)
				D12 L (%)	Definitive erythropoiesis D14 L (%)	
Δε::β^m^ wt LCR	166	1	99.5 ± 9.0	82.8 ± 11.9	83.0 ± 19.0	84.9 ± 11.0
Δε::β^m^ ΔHS3	44	1	79.7 ± 7.9	64.3 ± 1.7	61.2 ± 7.7	60.6 ± 3.4
	61	1	24.3 ± 0.7	76.6 ± 10.8	69.7 ± 4.3	67.5 ± 1.0
	69	2	58.9 ± 9.8	67.4 ± 8.1	57.2 ± 2.8	64.3 ± 6.4
Δε::β^m^ ΔHS3c	47	1	67.1 ± 20.8	84.6 ± 9.5	90.7 ± 6.4	103.0 ± 24.4
	81	2	41.1 ± 9.7	101.9 ± 9.9	99.7 ± 22.2	103.9 ± 11.9
wt β-YAC average		3	0.0	54.4 ± 6.60	67.4 ± 9.1	94.1 ± 2.3

## DISCUSSION

Although the human β-globin LCR may function as a holocomplex within an active chromatin hub, we provide evidence that within the aggregate hypersensitive site activation domain of the holocomplex, the individual HSs mediate preferential activation of specific globin genes during development. As a model to test this hypothesis we chose to mutate LCR 5′HS3 in conjunction with a globin gene order alteration (replacement) within the context of a human β-globin locus YAC. Several previous papers clearly identified the phenotypes associated with 5′HS3 deletion mutant β-YAC transgenic mice (Supplementary Table S1). A 2.3-kb complete deletion of 5′HS3 (Δ5′HS3) in β-YAC transgenics resulted in small decreases in ε- and γ-globin gene expression, with essentially normal β-globin gene expression (28), a relatively mild expression phenotype. A 225-or 234-bp deletion of the 5′HS3 core (Δ5′HS3c) in β-YAC transgenics abrogated ε-globin gene expression during primitive erythropoiesis, without affecting γ-globin gene expression at this developmental stage (23,30). During fetal liver definitive erythropoiesis, no to very low γ-globin expression was observed, but β-globin expression was unaffected. However, β-globin synthesis suffered from position effect variegation in the adult stage of definitive erythropoiesis. In spite of the similarity of the phenotypes between the large and small deletions, the severity was markedly amplified in the smaller core deletion mice. That is, the smaller 5′HS3c deletion was more catastrophic on gene expression than the larger 5′HS3 deletion. Addition of the −117 ^A^γ-globin Greek HPFH mutation to the 234 bp Δ5′HS3c β-YAC resulted in a phenotype indistinguishable from the 234 bp Δ5′HS3c transgenics (49). Singly mutant Greek HPFH β-YAC mice were previously shown to exhibit a strong hereditary persistence of fetal hemoglobin (HPFH) phenotype in adult mice (50). However, this HPFH point mutation had no effect on moderating the negative effect of the Δ5′HS3c mutation on γ-globin expression in transgenic mice. There was no γ-globin observed in adult mice even with the HPFH mutation, highlighting the importance of the 5′HS3 core sequences in regulating γ-globin expression. Finally, mutation of one of the seven GT motifs in the 225-bp 5′HS3 core, the GT6 motif, reduced the expression of the ε- and γ-globin genes during embryonic erythropoiesis (36). γ-globin gene expression was significantly reduced during fetal definitive erythropoiesis, but β-globin gene expression was not affected. Thus, the 5′HS3 GT6 motif is required for normal ε- and γ-globin transcription in the yolk sac and for γ-globin transcription in the fetal liver. We concluded that mutation of a single transcriptional motif in the LCR can have profound effects on gene expression. Importantly, we observed a general conservation of phenotype associated with a Δ5′HS3, deletion of the core element only or mutation of a single transcriptional motif within the core.

The lack of ε-globin expression in the Δ5′HS3c β-YAC transgenic mice suggested that 5′HS3 sequences of the LCR are involved directly in ε-globin gene activation. This reduction of ε-globin gene transcription in Δ5′HS3 or Δ5′HS3c β-YAC transgenics can be explained by two hypotheses. The first hypothesis posits that within the LCR holocomplex or ACH, the major determinant of LCR-globin gene interaction is LCR HS site specificity; i.e. for each globin gene, a specific HS or subset of the HSs is required for gene activation and the others are dispensable. During embryonic erythropoiesis, the interaction between the LCR and the ε-globin gene promoter involves specific sequences of 5′HS3 and specific sequences of the ε-globin gene promoter. When 5′HS3 or its core is deleted, these interactions do not take place and ε-globin gene transcription is diminished (28,30). 5′HS3 is not required for γ-globin transcription in the primitive yolk sac, but is necessary in the fetal definitive liver (30). Thus, LCR5′HS3 shows specificity for activation of ε-globin during primitive erythropoiesis and for γ-globin during fetal definitive erythropoiesis.

**Figure 9.**
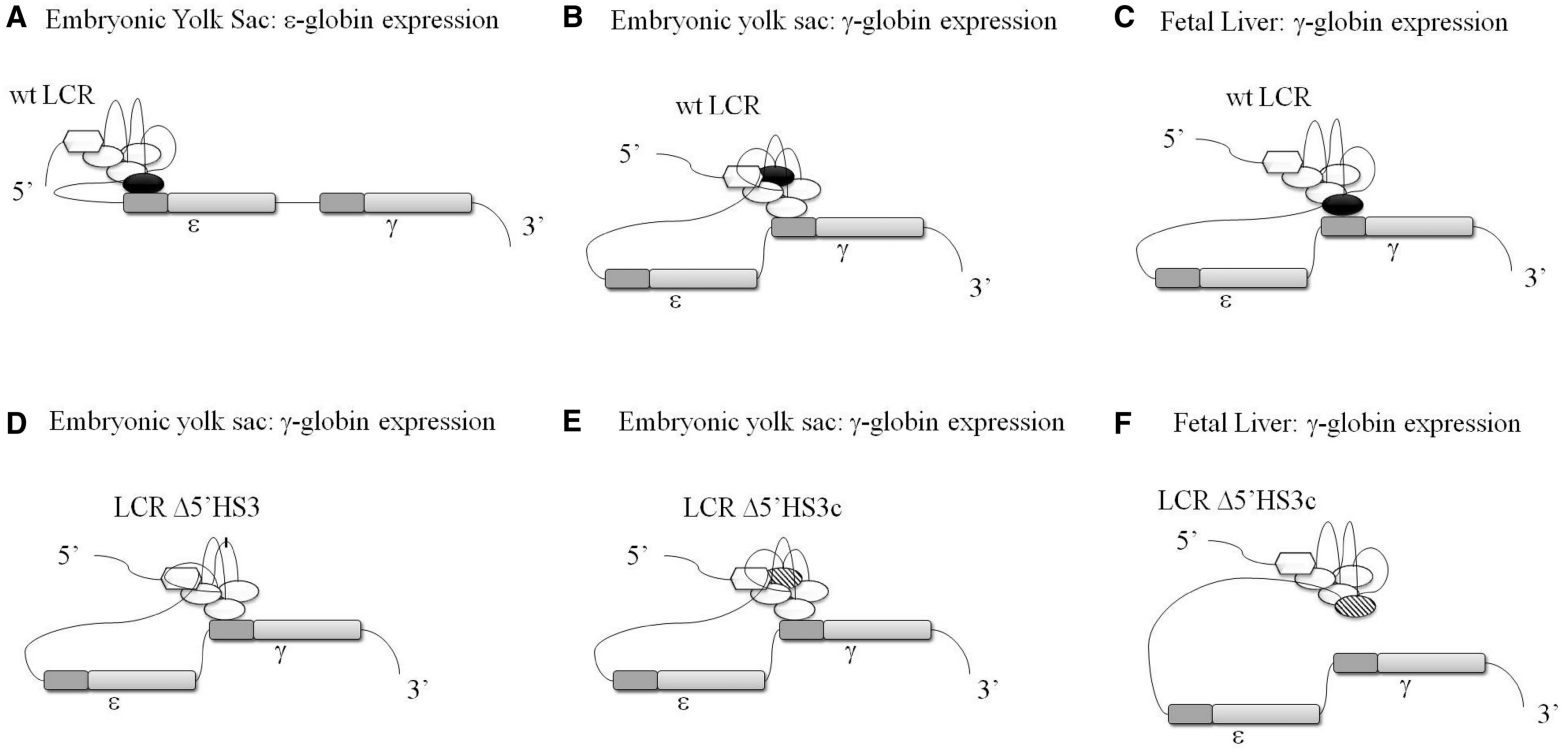
Model for LCR 5′HS3 gene activation specificity. These illustrations emphasize the interaction of 5′HS3 with a specific globin gene at each developmental stage. Panels A–C represent the interaction of the intact wt LCR with the ε- and γ-globin genes during primitive erythropoiesis (panels A and B) and fetal definitive erythropoiesis (panel C). Panels D–F represent the interaction of the 5′HS3 mutant LCRs with the ε- and γ-globin genes during primitive erythropoiesis (panels D and E) and fetal definitive erythropoiesis (panel F). Panel D shows the effect of the Δ5′HS3 on ε- and γ-globin during primitive erythropoiesis; panels E and F show the effect of the 5′ΔHS3c on these two genes during primitive erythropoiesis and fetal definitive erythropoiesis, respectively. For each panel, the developmental stage and globin gene are indicated at the top. The intact 5′HS3 is shown as a black oval (panels A–C), the complete 5′HS3 deletion (Δ5′HS3) is indicated by a missing oval (panel D) and the Δ5′HS3c is displayed as a hatched oval (panels E and F). The ε- and γ-globin genes are shown as rectangles; the darker color shade for each gene represents the promoter. (**A**) In the embryonic yolk sac, LCR 5′HS3 is essential for activation of ε-globin gene expression. (**B**) LCR 5′HS3 is not required for interaction with the γ-globin genes; another LCR 5′HS may be necessary. (**C**) In the fetal liver, LCR 5′HS3 is essential for activation of γ-globin gene expression. For the complete 5′HS3 and 5′ΔHS3c deletions [(**D**) and (E), respectively], γ-globin is expressed normally in yolk sac because the 5′HS3-mutated LCRs cannot interact with the ε-globin gene. However, LCR 5′HS3 is required for γ-globin expression during fetal definitive hematopoiesis (**F**), and in the absence of the 5′HS3 core region, γ-globin levels are markedly reduced. Altogether, this model suggests that HS site-specificity for gene activation is an important determinant of correct developmental expression of the β-like globin genes.

The second hypothesis states that the conformation of the LCR in the ACH is the most important determinant of LCR–globin gene interaction. If this hypothesis is true, than in the embryonic stage, the LCR would be expected to adopt a three-dimensional conformation that favors interaction with the first gene in the complex, the ε-globin gene. Consistent with this hypothesis, following the first switch from ε-globin gene expression in the primitive yolk sac to γ-globin gene expression in the fetal liver, the LCR would be predicted to assume an alternate conformation favorable to γ-globin activation. When 5′HS3 is deleted, an alternate conformation is assumed that decreases the chance that there will be an interaction between the LCR and the ε-globin gene. However, in 5′ΔHS3c mice, the next genes in locus, the γ-globin genes, are expressed (30). Thus, we assume that the LCR must still interact with the γ-globin genes during primitive erythropoiesis. Although γ-globin gene expression is normal during primitive erythropoiesis in these mutant mice, expression is extinguished during the fetal stage of definitive erythropoiesis, in contrast to mice carrying a normal β-YAC construct. These data suggest that a conformational change occurs in the Δ5′HS3c LCR during the switch from embryonic to definitive erythropoiesis, from one that supports γ-globin gene expression to one that does not (Figure 9). Alternately, the embryonic *trans*-acting environment may allow the mutant LCR to interact with and activate the γ-globin genes, but the fetal *trans*-acting environment may not support this interaction in the absence of the 5′HS3 core.

To distinguish between these two hypotheses, β-YAC lines were produced in which the ε-globin gene was replaced with a second marked β-globin gene (β^m^), coupled to an intact LCR, a 2.9-kb 5′ΔHS3 or a 234-bp 5′ΔHS3c (Figure 1). Δ5′HS3c Δε::β^m^ β-YAC mice expressed β^m^-globin throughout development beginning at Day 10 in the yolk sac (Figure 2A). γ-globin was expressed in the embryonic yolk sac, but at a reduced level in the fetal liver compared with transgenic lines containing an unmodified LCR (Figures 5A and 7A; Tables 1 and 2). Some wt β-globin was expressed in addition to β^m^-globin in adult mice, but at much reduced level compared with β^m^-globin (Table 3), which is probably due to the proximity of the β^m^-globin to the LCR, as demonstrated previously (46). The γ-globin phenotype is consistent with published data on Δ5′HS3c β-YAC mice (30).

Although ε-globin was not expressed in Δ5′HS3c β-YAC mice, β^m^-globin inserted in its place was expressed in Δ5′HS3c Δε::β^m^ β-YAC embryos, demonstrating that the 5′HS3 core was necessary for ε-globin expression during embryonic erythropoiesis, but not for β^m^-globin expression, nor for γ-globin. In the 12-day fetal liver, expression of γ-globin was reduced in both LCR mutants compared with constructs with unmodified LCRs, but that result could be attributed to gene competition between the β- and γ-globin promoters for interaction with the LCR (51). Interestingly, β-globin expression was higher in the Δ5′HS3c Δε::β^m^ β-YAC lines throughout development than with a full deletion of the 5′HS3 in the Δ5′HS3 Δε::β^m^ β-YAC construct, suggesting that the HS3 core deletion made the LCR even more permissive for β-globin expression (Figure 7B). LCR HS structure has been implicated in directing specificity of globin gene expression by alteration of DNA conformation at the HSs (52), which may explain the striking difference between the ε- and β-globin expression levels when the two genes are at the exact same location. The core deletion has an effect only on ε-globin expression in the yolk sac, whereas β-globin expression is largely unaffected by the core and entire 5′HS3 deletions.

If the LCR holocomplex conformation was the major determinant of LCR-mediated β-like globin gene activation, then the β^m^-globin gene should not have been expressed in transgenic mice with a deletion of 5′HS3 or the 5′HS3 core, similar to previous data with 5′HS3 mutations (28,30). We present evidence for a site specificity model of direct LCR HS–globin gene interaction with the confines of the holocomplex, where the holocomplex defines the three-dimensional structure of the open locus, but the specific LCR HS–gene interaction is the final determinant of temporal and spatial globin gene expression.

Our results, in combination with previous studies of human β-globin locus transgenic mice, contrast with similar analyses of the endogenous mouse β-globin locus, in which LCR 5′HSs were deleted (24,26,32,38,53,54). For example, and relevant to our study, a 5′HS3 deletion reduced the overall expression of the locus, although it preferentially decreased β^min^-globin expression over β^maj^-globin expression (53). HS site specificity for globin gene activation has not been observed in the murine locus; deletion of any of the individual HSs results in a phenotype similar to that for 5′HS3. This fundamental difference between the human and mouse loci is not readily explained, given that the HS core sequences of the human and mouse β-globin LCRs are highly conserved and the loci are similarly organized. Arguments against the human β-YAC transgenic data supporting the model presented above have been based on ectopic genome location of the transgene, coupled with possible resultant position effect variegation (PEV) of the transgene. When PEV was observed, a 150-kb β-YAC transgene was employed (55), whereas we have used a 248-kb (now more accurately known to be 213 kb) β-YAC containing more extensive locus-flanking sequences. Our use of the larger β-YAC in this and many other studies, as well as by others, over the last two decades has obviated PEV as an explanation for our results (56,57). We further minimize any chance for PEV by analyzing mice with complete locus copies that usually include extensive locus-flanking 5′- and 3′-sequences (Supplementary Figures S1–S3) and using multiple lines per construct. In our experience, controlling these two variables, coupled with the larger size of the YAC transgene, we have avoided or overcome PEV associated with the smaller YAC transgene. Regardless of the LCR mutation, our mice have consistently shown site-independent, copy-number-dependent expression whether the LCR is intact, carries a HS deletion or bears a simple mutation in the HS (28,30,36,49, this study). Our data occasionally show an outlier, such as the Δε::β^m^ Δ5′HS3c line 61 on Day 10 yolk sac sample (Table 4). However, this line showed expression consistent with the other two lines at all other developmental time points and for all globin genes assessed. Thus, for this mutant β-YAC, PEV was not observed for any of the genes within the locus, nor at any developmental stage. In this report, an additional control was built in by virtue of the β- for ε-globin gene swap in the constructs. For example, Δ5′HS3 mutant LCR function is normal regarding γ-globin gene expression, but not ε-globin gene expression, during primitive erythropoiesis (28,30) and the negative phenotype associated with ε-globin was reversed when β-globin was inserted in its place (this study). PEV does not account for the sum of all of these experimental outcomes; a more plausible explanation for our data is HS site specificity for globin gene activation, as our work posits. Therefore, our studies, when added to the data of previously published work, make a strong case for HS site specificity for globin gene activation. As such, these results may reflect inherent differences as to how the LCR functions within the human and mouse β-globin loci. These findings wait testing in genetically modified human progenitor cell systems or other human model systems.

One interpretation of these contrasting results in human versus mouse may be explained by taking into account the existence of two LCR functions, chromatin-opening activity and expression-enhancing activity. Although the mouse locus does not appear to require chromatin-opening activity, integrated human locus transgenes may require this activity in order to perform expression-enhancing activity. The 5′ΔHS3c might suppress the chromatin-opening activity, but the full-size deletion might have less influence on this activity. Integration of a wt, a core deletion or a full deletion transgene at a single genomic location to measure activity at the same single site might verify this possibility and allow understanding the mechanism.

## SUPPLEMENTARY DATA

Supplementary Data are available at NAR Online: Supplementary Table 1 and Supplementary Figures 1–4.

## FUNDING

National Institutes of Health (NIH) [DK053510, HL067336, DK081290 to K.R.P.]. Funding for open access charge: NIH [DK081290].

*Conflict of interest statement*. None declared.

## Supplementary Material

Supplementary Data
