# Supplementary material for: LCR 5′ hypersensitive site specificity for globin gene activation within the active chromatin hub
Source: Nucleic Acids Res. 2012 Oct 5;40(22):11256–69. doi: 10.1093/nar/gks900 (PMC3526258; doi:10.1093/nar/gks900)
Supplement: Supplementary Data [file supp_40_22_11256__index.html]

LCR 5′ hypersensitive site specificity for globin gene activation within the active chromatin hub — LCR 5′ hypersensitive site specificity for globin gene activation within the active chromatin hub — Supplementary Data 

# LCR 5′ hypersensitive site specificity for globin gene activation within the active chromatin hub

## Supplementary Data

files

**Files in this Data Supplement:**

- Supplementary Data - pdf file
