# Supplementary material for: LCR 5′ hypersensitive site specificity for globin gene activation within the active chromatin hub
Source: Nucleic Acids Res. 2012 Oct 5;40(22):11256–69. doi: 10.1093/nar/gks900 (PMC3526258; doi:10.1093/nar/gks900)
Supplement: Supplementary Data [file supp_gks900_nar-00920-m-2012-File004.pdf]

Table S1. Phenotypes of LCR 5'HS3 Deletion & Mutant YACs

Size of deletion	System used	Phenotype	Reference
2.3 Kb	213 Kb β-YAC	Small changes in ε and γ expression; essentially normal β expression.	28
225, 234 bp core	155, 213 Kb β-YAC	No ε, but γ in embryo; some or no γ, but β in fetus; β position effects in adult.	23, 30
234 bp core	213 Kb -117 HPFH β-YAC	Similar to Δ5'HS3 core β-YACs, no γ in adult even with HPFH mutation.	49
$GT6^{mut}$	213 Kb β-YAC	Decreased ε and γ in embryo; markedly reduced γ in fetus.	36

Figure S1

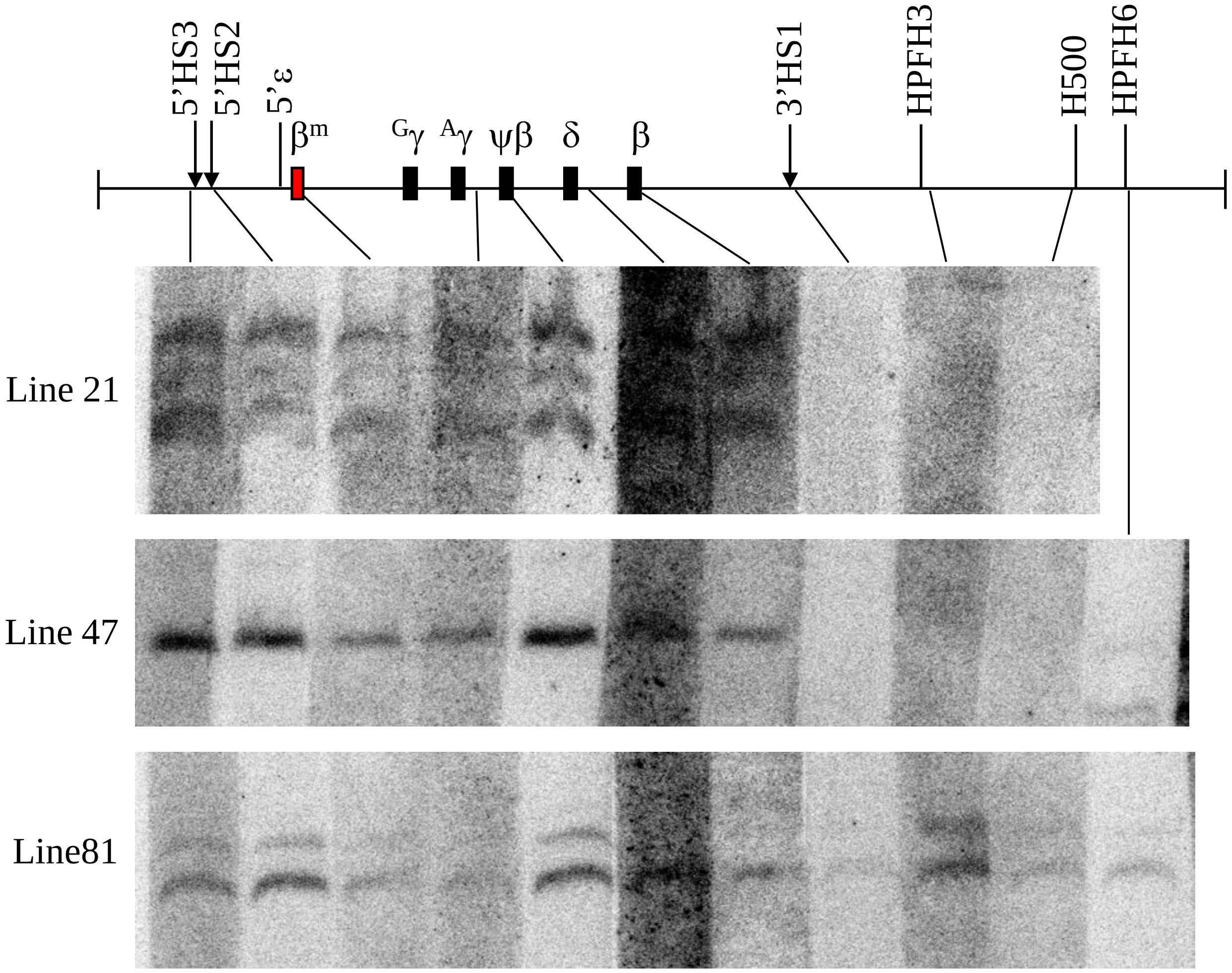

5'HS3
5'HS2
5'ε
βm
Gγ
Aγ
ψβ
δ
β
3'HS1
HPFH3
H500
HPFH6
Line 21
Line 47
Line81

Figure S2

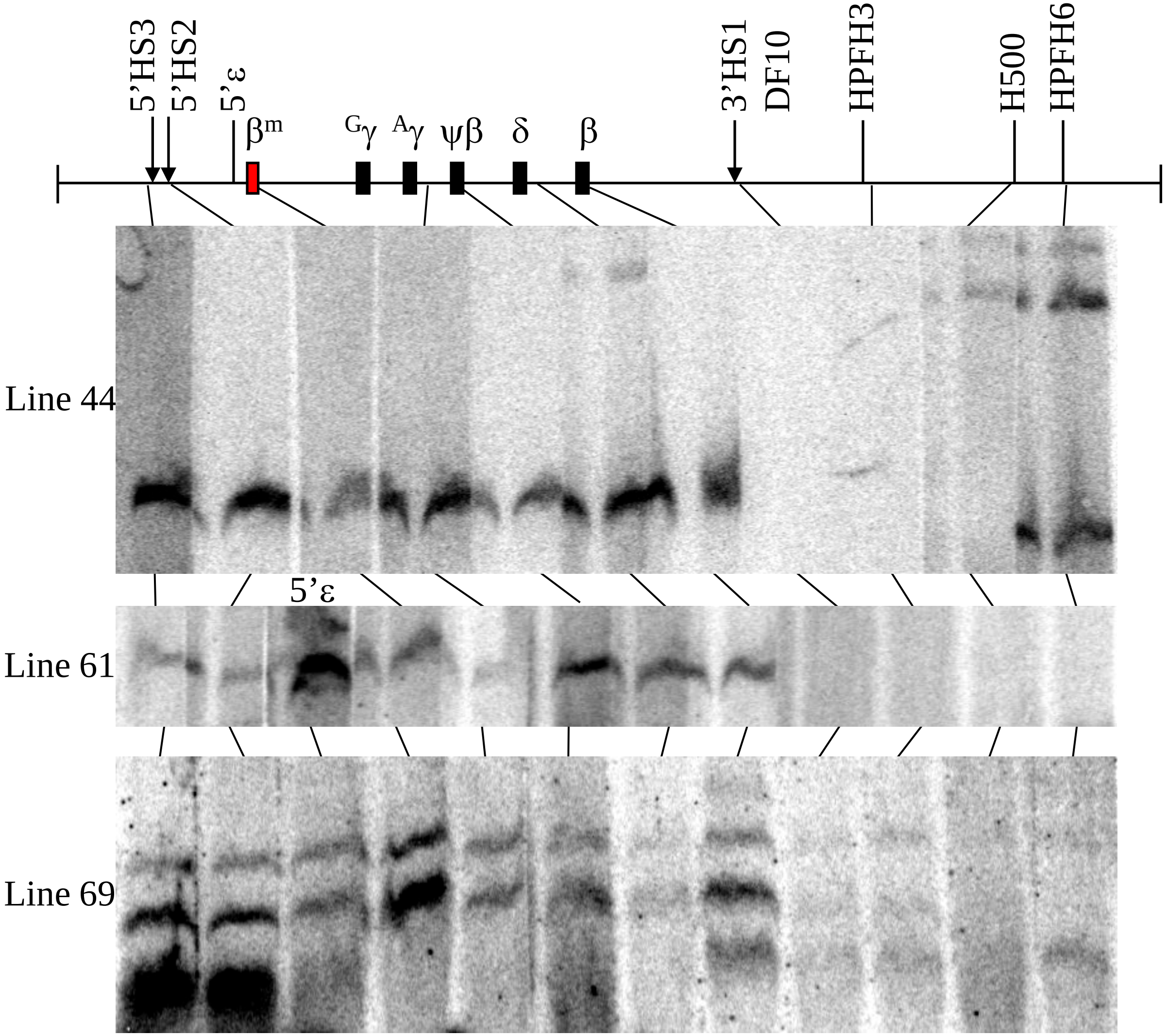

5'HS3
5'HS2
5'ε
βm
Gγ
Aγ
ψβ
δ
β
3'HS1
DF10
HPFH3
H500
HPFH6
Line 44
5'ε
Line 61
Line 69

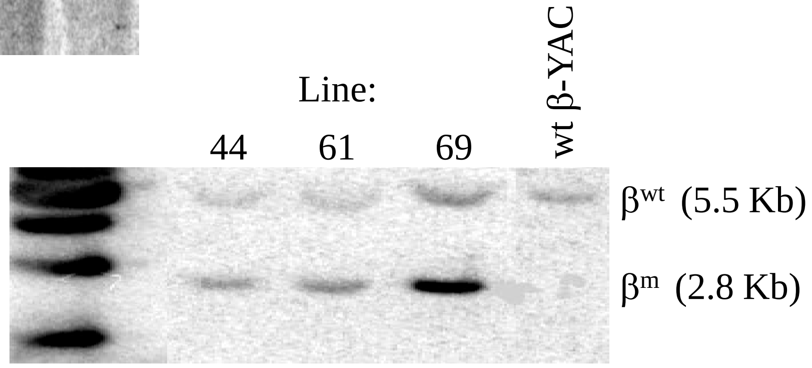

Line:
44
61
69
wt β-YAC
βwt (5.5 Kb)
βm (2.8 Kb)

Figure S3

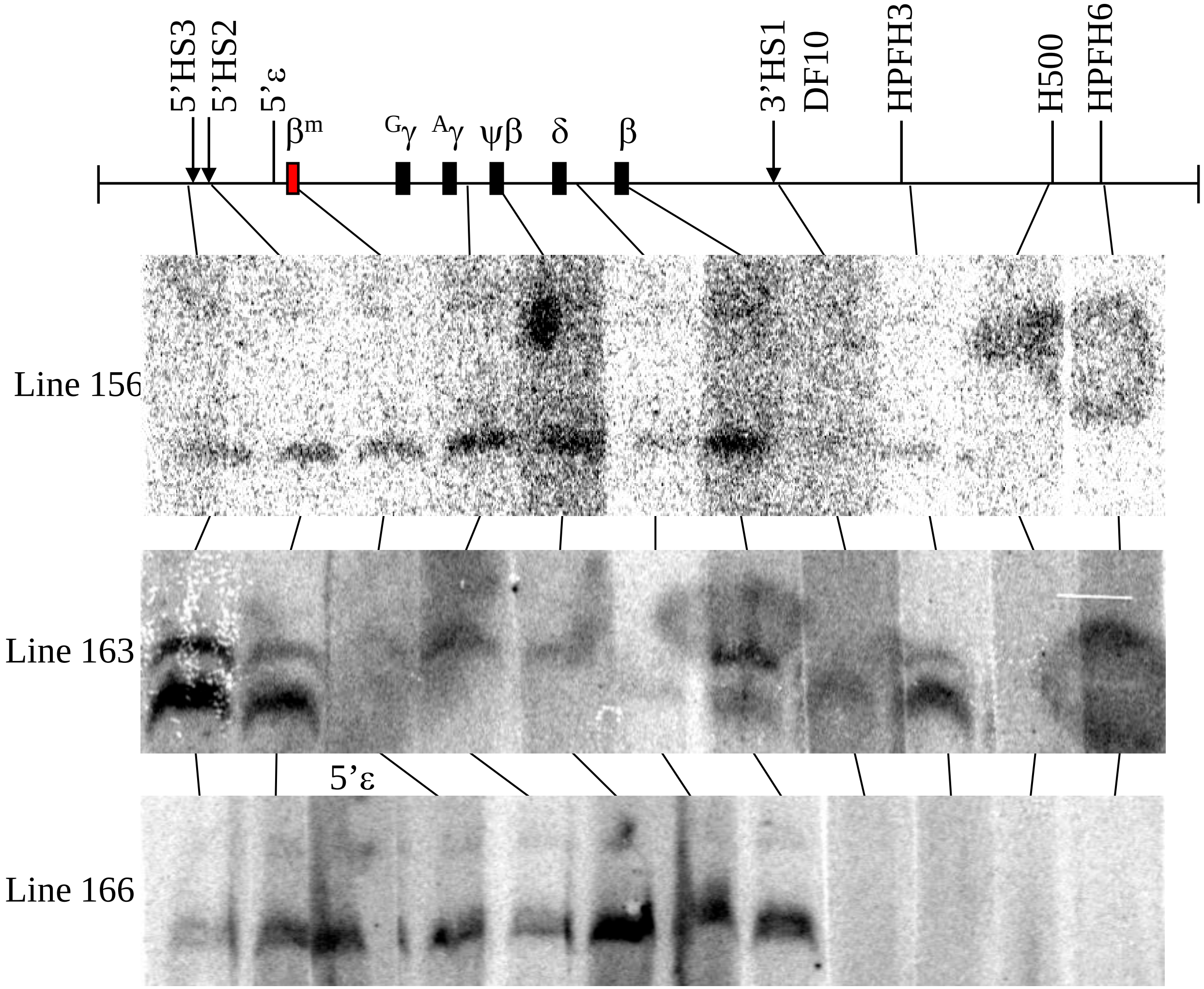
5'HS3
5'HS2
5'ε
βm
Gγ
Aγ
ψβ
δ
β
3'HS1
DF10
HPFH3
H500
HPFH6
Line 156
Line 163
5'ε
Line 166

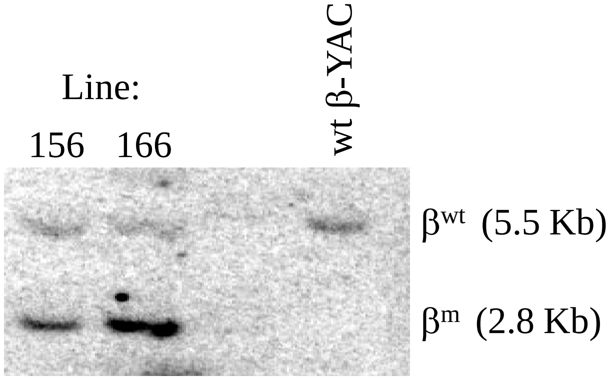
Line:
156
166
wt β-YAC
βwt (5.5 Kb)
βm (2.8 Kb)

Figure S4

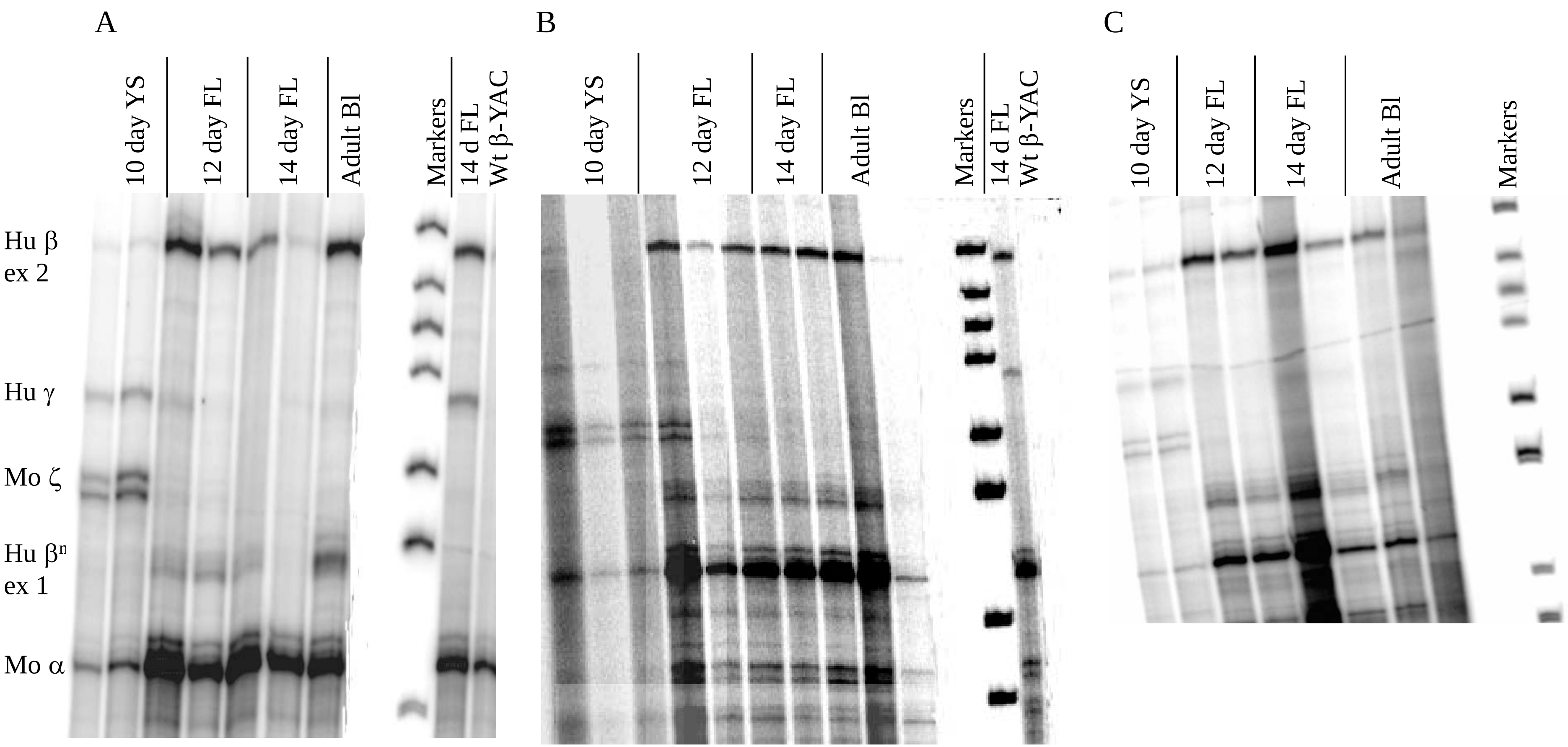

A
B
C
10 day YS
12 day FL
14 day FL
Adult Bl
Markers
14 d FL Wt β-YAC
Hu β ex 2
Hu γ
Mo ζ
Hu β[n] ex 1
Mo α

SUPPLEMENTAL FIGURE LEGENDS

Supplemental Figure 1. Structural analysis of Δ5'HS3c $\Delta\varepsilon::\beta^{m}$ β-YAC transgenes. A schematic diagram of the diagnostic 115 Kb *Sfi*I fragment contained within the β-YAC encompassing most of the β-globin locus is shown at the top of the figure. Autoradiographs of lines 21, 47 and 81 are displayed. Analysis was performed as described in Materials and Methods (46). The probes used are listed above each autoradiograph. Line 21 contains three deleted copies of the Δ5'HS3c $\Delta\varepsilon::\beta^{m}$ β-YAC, but each copy contains an intact β-globin locus. Line 47 contains one deleted copy of the of β-YAC that contains an intact β-globin locus. Line 81 contains two intact copies of the YAC, both larger than 115 Kb. Copy numbers were validated by standard Southern blot analysis. Additional Southern blot hybridizations confirmed the presence of the 3' β-globin gene enhancer in copies that are truncated downstream of the β-globin gene. Previous experience has demonstrated that if the locus on the YAC is intact from the LCR through this enhancer, globin gene expression is normal. We also demonstrated the presence of both the marked β (β^{m})- and wild-type β (β^{wt})-globin genes and the integrity of 5'HS5 and 4 in all three lines (data not shown). For the analyses shown in this figure and Supplemental Figures 2 and 3, the 5'HS3 probe flanks both the 5'HS3 and 5'HS3c deletions.

Supplemental Figure 2. Structural analysis of Δ5'HS3 $\Delta\varepsilon::\beta^{m}$ β-YAC transgenes. The figure layout is as described in the legend to Supplemental Figure 1. Line 44 contains one copy of the β-YAC extending from 5'HS3 through the β-globin gene and downstream sequences. Two other copies span the H500-HPFH6 region. These latter two fragments do not contribute to β-like globin gene expression. Line 61 carries one copy of the transgene spanning 5'HS3 through the β^{wt}-globin gene. Line 69 has two copies of the β-YAC with sequences from 5'HS3 through the

HPFH3 breakpoint, a third deleted copy containing only 5’HS3 and 2, and a fourth deleted copy extending from 3’HS1 through the HPFH6 breakpoint. Additional analyses demonstrated the presence of both the β^{m}- and β^{wt}-globin genes (smaller panel), β^{wt}-globin enhancer sequences and LCR 5’HS5 and 4 in all lines (data not shown).

Supplemental Figure 3. Structural analysis of wt LCR $\Delta\varepsilon$::β^{m} β-YAC transgenes. The figure layout is as described in the legend to Supplemental Figure 1. Three lines (156, 163 and 166) carry at least one β-YAC extending from 5’ HS3 through the β-globin gene. Line 156 has one *Sfi*I fragment extending from 5'HS3 through the HPFH3 breakpoint. Line 163 has three copies, one extending from 5'HS3 through the β-globin gene, one fragment encompassing only 5’HS3 and 2, and a second fragment extending from the δ-globin gene through the HPFH3 breakpoint. Line 3 has one copy spanning 5'HS3 through the β-globin gene. Additional analyses demonstrated the presence of both the β^{m}- and β^{wt}-globin genes for lines 156 and 166 (smaller panel), as well as β^{wt}-globin enhancer sequences and LCR 5’HS5 and 4 in all lines (data not shown).

Supplemental Figure 4. Effect of LCR mutations on β-like globin transcription in Δ5’HS3c, Δ5’HS3, and wt LCR $\Delta\varepsilon$::β^{m} β-YAC transgenic mice. RPA was performed as referenced in Materials and Methods. Labeling is as shown in the legend to Figure 2, except sizes are not shown for protected fragments.
